# Change in left ventricular function and outcomes following high-risk percutaneous coronary intervention with Impella-guided hemodynamic support

**DOI:** 10.3389/fcvm.2024.1416613

**Published:** 2024-07-05

**Authors:** Serdar Farhan, Michael Freilich, Gennaro Giustino, Birgit Vogel, Usman Baber, Samantha Sartori, Haroon Kamran, Roxana Mehran, George Dangas, Prakash Krishnan, Annapoorna Kini, Samin K. Sharma

**Affiliations:** ^1^ The Zena and Michael A. Wiener Cardiovascular Institute, Icahn School of Medicine at Mount Sinai, New York, NY, United States; ^2^ Moses Division, Department of Internal Medicine, Montefiore Medical Center, Bronx, NY, United States

**Keywords:** high-risk percutaneous coronary interventions, Impella, left ventricular ejection fraction, mechanical circulatory devices, SYNTAX score

## Abstract

**Introduction:**

High-risk percutaneous coronary interventions (HRPCI) are a potential treatment option for patients with reduced left ventricular ejection fraction (LVEF) and coronary artery disease. The extent to which such intervention is coupled with improvement in LVEF and associated with favorable outcomes is unknown.

**Methods:**

We aimed to characterize the incidence and correlates of LVEF improvement after Impella-guided HRPCI, and compare clinical outcomes in patients with versus without LVEF improvement. Data on consecutive patients undergoing Impella-guided HRPCI from a single center registry were analyzed. LVEF-improvement was defined as an absolute increase of LVEF of ≥10% measured at ≥30‐days after intervention. The primary outcome was a composite of all‐cause death, myocardial infarction or target vessel revascularization within 1-year.

**Results:**

Out of 161 consecutive patients undergoing Impella-guided HRPCI from June 2008 to December 2017, 43% (*n* = 70) demonstrated LVEF-improvement (baseline LVEF of 25.09 ± 6.19 to 33.30 ± 11.98 post intervention). Patients without LVEF-improvement had higher frequency of previous MI (61.5% vs. 37.1%, *p* = 0.0021), Q-waves on ECG (17.6% vs. 5.7%, *p* = 0.024) and higher SYNTAX scores (30.8 ± 17.6 vs. 25.2 ± 12.2; *p* = 0.043). After correction of these confounders by multivariable analysis, no significant differences were found regarding the composite endpoint in patients with versus without LVEF-improvement (34.9% vs. 38.3%; *p* = 0.48).

**Discussion:**

In this single-center retrospective analysis, we report the following findings. First, LVEF improvement of at least 10% was documented in over 40% of patients undergoing Impella supported high-risk PCI. Second, a history of MI, Q-waves on admission ECG, and higher baseline SYNTAX scores were independent correlates of no LVEF improvement. Third, one year rates of adverse CV events were substantial and did not vary by the presence or absence of LVEF improvement Prospective studies with longer follow-up are needed to elucidate the impact of LVEF improvement on clinical outcomes.

## Introduction

Up to 20% of patients with complex coronary artery disease are deemed poor surgical candidates, leading this subset of the population to be underserved with regards to coronary revascularization (1). The reasons underlying this fact are multifaceted and can be traced to advanced age, multiple medical co-morbidities, left ventricular dysfunction, decompensated heart failure, among others (2). Such patients suffer a markedly higher rate of adverse outcomes, even if a percutaneous coronary intervention (PCI) is sought (1, 3). However, with recent advances in mechanical circulatory support-assisted PCI, the ability of improved clinical outcomes in this population remains a possibility. The added hemodynamic stability provided by these devices provides additional support not previously available.

There is scarce data available evaluating whether these interventions are associated with an improvement in left ventricular (LV) function and subsequent clinical outcomes. Findings from a randomized trial demonstrated that reverse LV remodeling occurred in 51% of patients undergoing high-risk PCI with Impella support, which was associated with a reduction in 30-day adverse events (4). However, the extent to which these benefits are generalizable to an unselected cohort and durable over longer-term follow-up remains unknown. Therefore, we aimed to investigate the correlates of LVEF change and the association between LVEF improvement and 1-year clinical events in patients undergoing Impella-supported high-risk PCI at our institution.

## Materials and methods

The study cohort was selected from a prospective registry maintained at Mount Sinai Heart. All patients who underwent Impella-supported PCI were selected for eligibility for inclusion in the present analysis.

### Inclusion and exclusion criteria and definitions

Between June 2008 and December 2017, a total of 328 patients underwent Impella 2.5® (Abiomed Inc. Danvers, Massachusetts) supported PCI at Mount Sinai Hospital. High-risk PCI was defined according to our institutional algorithm Complex PCI (Long calcified lesion, Bifurcation lesion, Unprotected LM lesion, SVG lesion) with concomitant LVEF >35%; Complex PCI or High SYNTAX score >32/STS risk for mortality >5% or extensive revascularization with concomitant LVEF 20%–35%; or simple or complex PCI or inoperable patient with concomitant LVEF <20% (Supplementary Figure S1). The inclusion criteria for the present analysis were (i) underlying CAD undergoing Impella-supported PCI (ii) LVEF measurement before and at least 30 days after the procedure. Patients who expired within index hospitalization and those with missing LVEF evaluation during follow-up were excluded. For the purpose of the present analysis, patients were grouped according to LVEF improvement of at least 10% (delta LVEF >10%) vs. less than 10% (delta LVEF <10%). LVEF was calculated either via the Simpson method using transthoracic echocardiogram or MUGA nuclear medicine scans by plotting red blood cell technetium using a gated ECG approach.

An institutional review board approved the study.

### Endpoints

The endpoint of interest was a composite of all-cause death, myocardial infarction (MI), or target vessel revascularization (TVR) within 1-year of follow-up. MI was defined according to the 3rd universal definition of MI (5) and TVR was defined according to the academic research consortium (ARC) (6). Follow-up information was captured via telephone calls by trained research coordinators at one year after index PCI. Source documents were obtained for those patients reporting any adverse events. All information was then forwarded to a clinical events committee for formal adjudication.

### Statistical analysis

Continuous variables are presented as mean ± SD. Categorical variables are presented as percentages. Chi-square test was used to compare differences between categorical variables. The independent-samples *t*-test was used to compare continuous variables with normal distribution, and the Mann-Whitney test was used to compare continuous variables without normal distribution. Crude 1-year event rates were calculated using the Kaplan-Meier method and a log-rank test to assess differences. A multivariate linear regression analysis with purposeful selection of variables was used to identify independent correlates of LVEF change (delta LVEF, calculated as the difference in LVEF measurement between follow-up and baseline).

## Results

**Figure 1:**
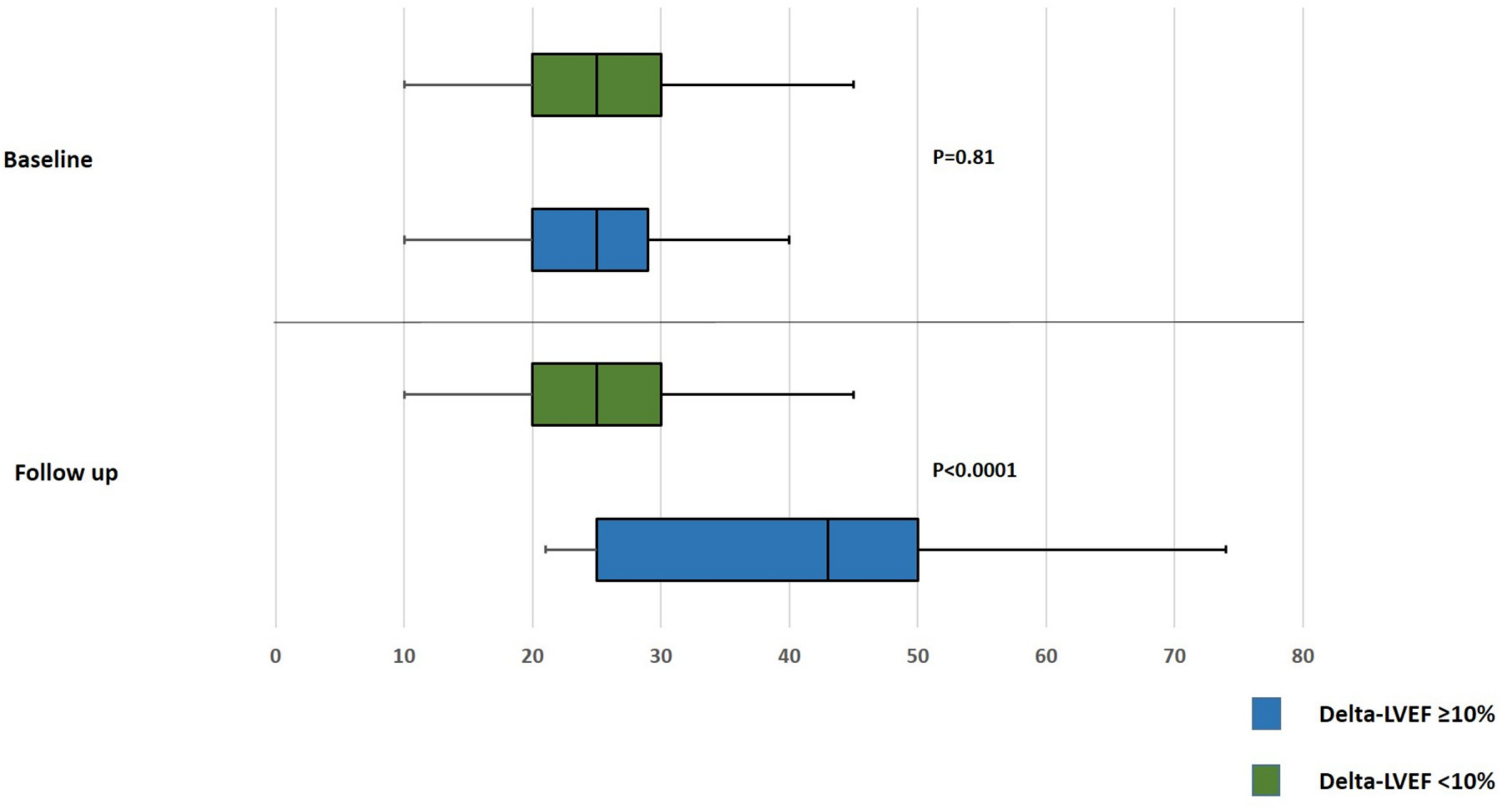
Box-whiskers plot for comparing baseline and follow up left ventricular ejection fraction after Impella-guided percutaneous coronary intervention. LVEF, left ventricular ejection fraction.

**Figure 2:**
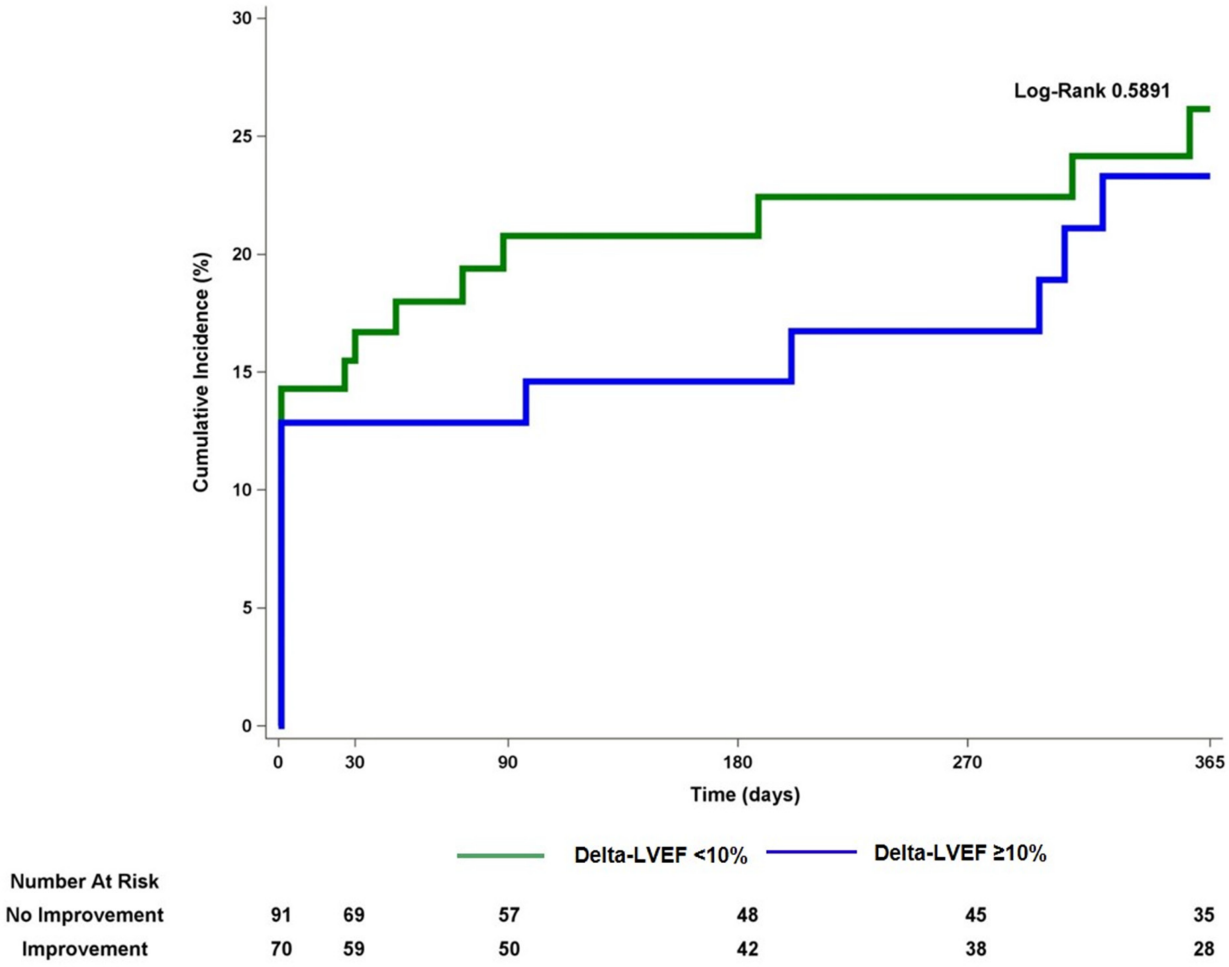
Kaplan-Maier curve for the composite endpoint of all-cause death, myocardial infarction or target vessel revascularization comparing patients with vs. without improvement in left ventricular ejection fraction. LVEF, left ventricular ejection fraction.

Out of 328 patient who underwent Impella-supported PCI a total of 161 eligible patients with baseline LVEF 25.1 ± 6.2 with a median follow-up of 112 days were included in the study. Baseline and procedural characteristics of patient included vs. not included in the analysis are presented in a Supplementary Table S1. Multivessel disease was present in 88.6% of patients. Baseline and procedural characteristics are presented in Tables 1, 2. LVEF improvement of greater than 10% was observed in 70 patients (43%). This group showed LVEF of 39.1 ± 11.2% vs. 24.5 ± 6.5% in the group without delta LVEF <10% (*p* ≤ 0.0001) (Figure 1 and Supplementary Figure S2). There were no significant differences between groups with regards to age, sex, cardiovascular risk factors, renal impairment, anemia, history of peripheral as well as cerebrovascular disease or clinical presentation. Upon further review, patients from the delta-LVEF <10% group showed a significantly higher prevalence of previous MI (61.5% vs. 37.1%, *p* = 0.0021) and Q waves on admission ECG (17.6% vs. 5.7%, *p* = 0.024). PCI was successful in 87.3% and multivessel PCI was performed in 52.5% of the study population. Procedural characteristics, including stent length, bifurcation lesion, severe calcification, and stent diameter were similar between groups. Furthermore, the SYNTAX score was significantly higher in patients from delta-LVEF <10% compared to delta-LVEF ≥10% group (30.8 ± 17.6 vs. 25.2 ± 12.2; *p* = 0.043) (Table 1). There was a non-significant trend for lower residual SYNTAX score in the delta-LVEF ≥10% group compared to delta-LVEF <10% (Table 1). Clinical outcomes are presented in Supplementary Table S2. There were no significant differences in the composite endpoint of death, MI, or TVR at 1-year (Figure 2). In a multivariable linear regression model, history of prior MI, Q-waves on admission ECG and higher baseline SYNTAX score were independent correlates of LVEF change (Table 2). There were no significant differences in the composite endpoint of all-cause death, MI, and TVR over one year between patients from delta-LVEF ≥10% and delta-LVEF <10% (34.9% vs. 38.3%, *p* = 0.481).

**Table 1:** Clinical and procedural characteristics of patients according to left ventricular function improvement.

	Delta-LVEF <10% 91 (57.0%)	Delta-LVEF ≥10% 70 (43.0%)	*P*-value
Age, years	67.6 ± 11.8	67.8 ± 12.5	0.92
Ethnicity Caucasian	43 (47.3%)	38 (54.3%)	0.55
Female sex	14 (15.4%)	16 (22.9%)	0.22
BMI (kg/m^2^)	26.23 ± 4.22	27.47 ± 5.11	0.09
Hyperlipidemia	82 (90.1%)	62 (88.6%)	0.75
Hypertension	83 (91.2%)	62 (88.6%)	0.57
Diabetes mellitus	44 (48.4%)	40 (57.1%)	0.26
CKD	40 (44.0%)	24 (35.3%)	0.27
Anemia	46 (50.5%)	41 (58.6%)	0.31
Current smoker	14 (15.4%)	6 (8.6%)	0.19
Ischemic history			
Previous MI	56 (61.5%)	26 (37.1%)	0.002
Previous CABG	15 (16.5%)	5 (7.1%)	0.07
PAD	11 (12.1%)	5 (7.1%)	0.29
Cerebrovascular disease	11 (12.1%)	11 (15.7%)	0.50
Presentation			
Stable Angina	36 (39.6%)	28 (40.0%)	0.95
Unstable angina	38 (41.8%)	21 (30.0%)	0.12
NSTEMI	9 (9.9%)	15 (21.4%)	0.041
STEMI	3 (3.3%)	3 (4.3%)	0.74
ECG results			
Bundle branch block	21 (23.1%)	18 (25.7%)	0.69
Q waves	16 (17.6%)	4 (5.7%)	0.02
ST changes	16 (17.6%)	17 (24.3%)	0.29
Atrial fibrillation/flutter	2 (2.9%)	2 (3.5%)	0.85
Lesion characteristics			
Lesion length	51.9 ± 30.4	47.7 ± 27.9	0.37
ISR	16 (17.6%)	11 (15.7%)	0.75
CTO	21 (23.1%)	9 (12.9%)	0.09
Bifurcation lesion	32 (35.2%)	27 (38.6%)	0.65
ACC/AHA type B2C lesion	87 (95.6%)	69 (98.6%)	0.28
Thrombotic	5 (5.5%)	10 (14.3%)	0.057
Calcification	31 (34.1%)	25 (35.7%)	0.91
PCI with stent	87 (95.6%)	68 (97.1%)	0.60
Stent length	51.5 ± 26.6	51.9 ± 30.1	0.93
Maximum stent diameter (mm)	3.50 (3.00–3.50)	3.50 (3.00–3.75)	0.80
Pre-TIMI 0 or 1	23 (25.3%)	14 (20.0%)	0.43
Post-TIMI 0 or 1	7 (7.7%)	1 (1.4%)	0.06
SYNTAX score	30 (17–40.5)	25 (16–32)	0.043
Residual SYNTAX score	8 (1–20)	5 (0–14)	0.088
PCI vessel			
LAD	62 (68.1%)	55 (78.6%)	0.14
LCx	40 (44.0%)	32 (45.7%)	0.82
RCA	24 (26.4%)	18 (25.7%)	0.92
LM	20 (22.0%)	16 (22.9%)	0.89
SVG	6 (6.6%)	2 (2.9%)	0.27
Procedure length (min)	110.6 ± 52.6	118.8 ± 64.2	0.37
Contrast volume (ml)	167.0 ± 61.5	173.6 ± 65.7	0.51
Number of lesions	2.00 (1.00–3.00)	2.00 (1.00–3.00)	0.58
Baseline LVEF	25.2 ± 6.6	24.9 ± 5.7	0.81
Follow-up LVEF	25.9 ± 6.8	42.9 ± 10.2	<0.0001
Delta LVEF	0.7 ± 6.2	18.0 ± 8.1	<0.0001

Mean ± SD or median (IQR). *N* (%).

BMI, body mass index; CABG, coronary artery bypass graft; CKD, chronic kidney disease defined as estimated glomerular filtration rate 60 ml/min; CTO, chronic total occlusion; ISR, in-stent restenosis; LAD, left anterior descending artery; LCx, Left circumflex artery; LM, left main; LVEF left ventricular ejection fraction; MI, myocardial infarction; NSTEMI, non-ST-elevation myocardial infarction; PAD peripheral artery disease; PCI, percutaneous coronary intervention; RCA, right coronary artery; STEMI, ST-elevation myocardial infarction; SVG saphenous vein graft.

**Table 2:** Linear regression analysis of correlates of left ventricular function improvement in patients undergoing Impella-guided high-risk percutaneous coronary intervention.

Variable	Beta coefficient	*P*-value
Prior MI (Yes vs. No)	−6.73	0.0009
Q Waves on admission ECG (Yes vs. No)	−5.91	0.0438
CKD (Yes vs. No)	−1.49	0.5037
Syntax score (per 5 units increase)	−0.83	0.0297
Baseline LVEF (per unit increase)	−0.18	0.2855
Lesion length (per unit increase)	−0.08	0.0854
Anemia (Yes vs. No)	−0.02	0.9927
Age (per unit increase)	0.04	0.7003
DM (Yes vs. No)	0.86	0.6744
Severely calcified lesion (yes vs. No)	1.14	0.6161
Presentation (Stable angina vs. ACS)	2.08	0.3536
Number of lesions (per lesion increase)	2.12	0.0742
Sex (Female vs. Male)	2.53	0.3372
PAD (Yes vs. No)	2.94	0.4588
ACC/AHA type B2 or C lesion (Yes vs. No)	10.95	0.0550

MI, myocardial infarction; ECG, electrocardiogram; ACC/AHA, American College of Cardiology/American Heart Association; LVEF, left ventricular ejection fraction; ACS, acute coronary syndrome; PAD, peripheral vascular disease; CKD, chronic kidney disease; DM, diabetes mellitus.

## Discussion

In this single-center retrospective analysis, we report the following findings: First, LVEF improvement of at least 10% was documented in over 40% of patients undergoing Impella supported high-risk PCI. Second, a history of MI, Q-waves on admission ECG, and higher baseline SYNTAX score were independent correlates of no LVEF improvement. Third, one-year rates of adverse CV events were substantial and did not vary by the presence or absence of LVEF improvement.

Patients with complex CAD and concomitant left ventricular dysfunction are usually characterized by significant comorbidity, thus rendering surgical revascularization prohibitive or very high risk, with high evidence of mortality noted (1). A meta-analysis of randomized clinical trials and registry studies comparing CABG vs. PCI vs. medical therapy in patients with CAD and LVEF ≤40% showed more favorable outcomes with surgical revascularization (3, 7). However, the majority included studies that did not utilize mechanical circulatory devices for the PCI group, resulting in higher rates of incomplete revascularization (3, 7). The introduction of mechanical circulatory support devices made such patients more amendable for PCI with complete revascularization (8–10). Recently, Burzotta et al. found an improvement of LVEF in about 70% of patients undergoing high-risk PCI by Impella support (11). Additionally, the authors found that completeness of revascularization measured by the British Cardiovascular Intervention Society (BCIS) Jeopardy Score (JS) was associated with improvements of LVEF and clinical outcomes at a mean follow-up of 14 months (11). Moreover, in a pooled analysis of the PROTECT II trial and the cVAD registry, Russo et al. showed that low baseline LVEF, absence of congestive heart failure, and the number of treated vessels were independent correlates of LVEF improvement (12). All-cause death in our study was 3.81% at 12 months as compared to 10.5% in the study of Burzotta et al. This difference might be attributed to the higher rate of acute coronary syndrome patients (73%) and higher rate of left main (LM) interventions (44%) in the study of Burzotta et al. (11) as compared to the present study.

Mechanical circulatory devices provide additional hemodynamic stability not previously available, allowing for the opportunity of complete revascularization. This was confirmed in Burzotta et al., where Impella-guided PCI resulted in a higher rate of complete revascularization (11). This proves to be important as a sub-study of the ACUITY trial showed complete revascularization measured by residual SYNTAX score was associated with improved 1-year outcomes, while a residual SYNTAX score of >8 was associated with poor prognosis (13). In the present study, residual SYNTAX score was slightly lower in LVEF improvement patients without reaching statistical significance. Furthermore, both a history of myocardial infarction and Q-waves on admission ECG were significant negative correlates of LVEF improvement in the present study. Both parameters indicate developed scar tissue, making an expectation of LVEF improvement less likely.

Previously the OAT trial showed no difference in the composite endpoint of all-cause death, re-infarction, or heart failure readmission when PCI was compared to medical management only in patients deemed high risk who were less than one month after an MI with a total occlusion of the infarcted artery (14). In an ancillary study, the authors found that myocardial viability was associated with the improvement of LVEF regardless of assigned treatment (15). The REVIVED trial showed no decrease in all-cause mortality or hospitalization for HF when comparing PCI plus optimal medical therapy vs. optimal medical therapy alone, in patient with LVEF ≤35% and extensive coronary artery disease (16). Similarly, others were also not able to document an association of LVEF restoration after revascularization with improved clinical outcome (17–19). However, sample size, mode of revascularization by surgery vs. PCI, the variability of the measured endpoints, and follow up duration might be the reason for the differing findings obtained as compared to the present investigation (4, 17–19). Improvement of clinical outcomes with revascularization over medical therapy became evident only after long-term follow-up, as highlighted in the extension of the STICH trial (20). Furthermore, the definition of LVEF improvement varied between the studies (4, 17–19). Our study showed similar results with regards to no improvement in clinical outcomes even in patients with significant LVEF improvement.

### Limitation

We are aware of several limitations of the present analysis. First, the data provided herein were derived from a single-center observational study, which limits the generalizability of our results. Furthermore, due to the retrospective design, several unmeasured confounders might have affected the results obtained in this analysis. Despite the encouraging results of the recently published DanGer Shock trial the present analysis excluded patients in shock. However, ongoing Randomized trials e.g., PROTCT IV are awaited to provide definitive answers on the impact of mechanical support device assisted high-risk PCI on changes in LVEF and subsequently on clinical outcomes. Second, we did not systematically evaluate preoperative scores e.g., the society of thoracic surgery mortality score and EURO-Score, providing additional opportunities for confounders. Furthermore, we did not evaluate imaging or hemodynamic parameters e.g., end-diastolic and end-systolic volumes, right ventricular function, and systolic pressure, which have been associated with better predictive value for outcomes after high-risk PCI (21, 22). Third, analyses with respect to clinical outcomes are underpowered, introducing the possibility of a type II error. Fourth, due to the small sample size of our study, our results are hypothesis-generating rather than conclusive. Larger studies such as the PROTCT IV trial would potentially address this issue. Fifth, our follow-up post intervention was only one year, and significant value would be derived in future studies with longer term follow-up. Furthermore, such interventions might have an impact on quality of life measures. However, this prospective registry did not include such metrics during follow up which is a limitation of the present study. Finally, despite obtaining stress test for all patients (excluding those with NSTEMI and STEMI) before the procedure, systematic viability testing was not performed. Adding viability testing might have impacted our findings.

## Conclusion

History of MI, Q-waves on admission ECG and higher SYNTAX score were negative correlates of LVEF improvement in patients undergoing Impella-guided high-risk PCI. An increase of LVEF did not translate into an improvement of clinical outcomes in this patient population. Further research is warranted to elucidate predictors of LVEF improvement and their impact of on clinical outcomes in patients with ischemic heart disease undergoing high-risk intervention.

## Data Availability

The datasets presented in this article are not readily available because only people on IRB had access to this data and is under strict protection by Mount Sinai Hospital. Requests to access these datasets should be directed to serdar.farhan@mountsinai.org.
