# Supplementary material for: Change in left ventricular function and outcomes following high-risk percutaneous coronary intervention with Impella-guided hemodynamic support
Source: Front Cardiovasc Med. 2024 Jul 5;11:1416613. doi: 10.3389/fcvm.2024.1416613 (PMC11258011; doi:10.3389/fcvm.2024.1416613)

Supplementary Figure 1:

Proposed algorithm for utilization of Impella-Supported PCI in patients undergoing percutaneous coronary intervention.

Supplementary figure 2:

Line diagram of baselines and follow up left ventricular ejection fraction in patients undergoing Impella-Supported percutaneous coronary intervention.

Supplementary Figure 1

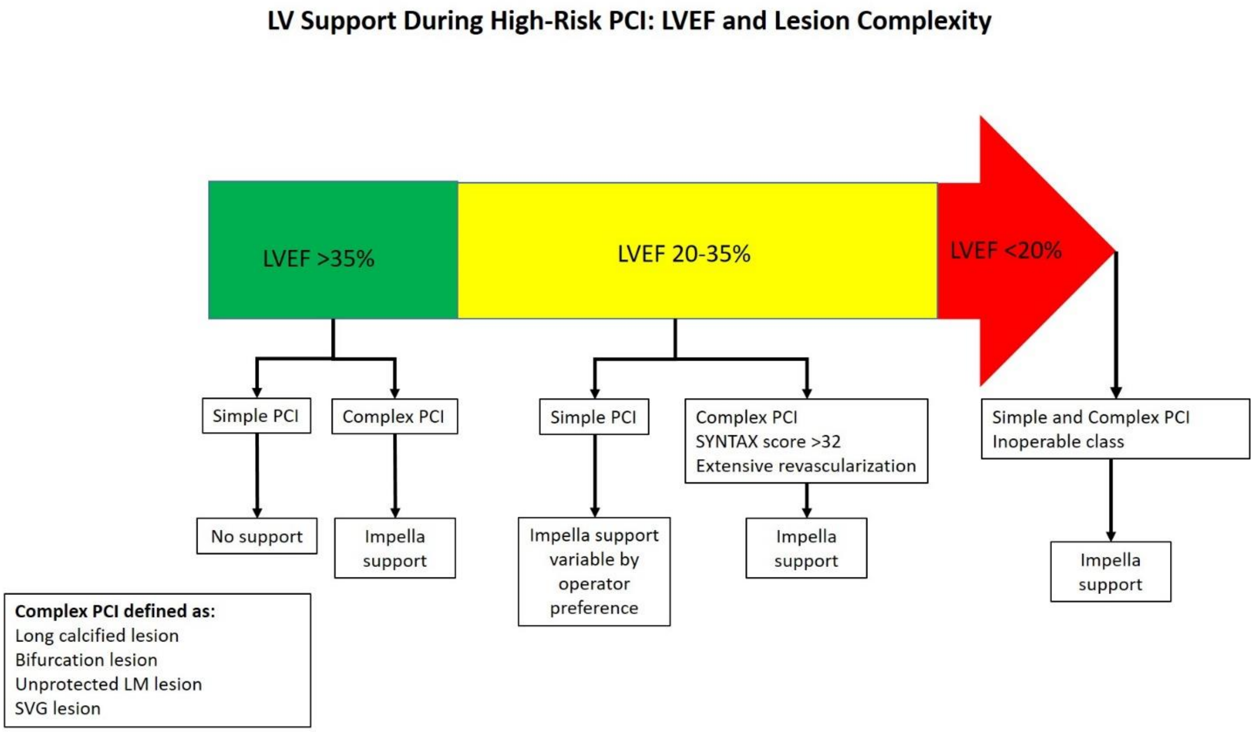

Supplementary Figure 2

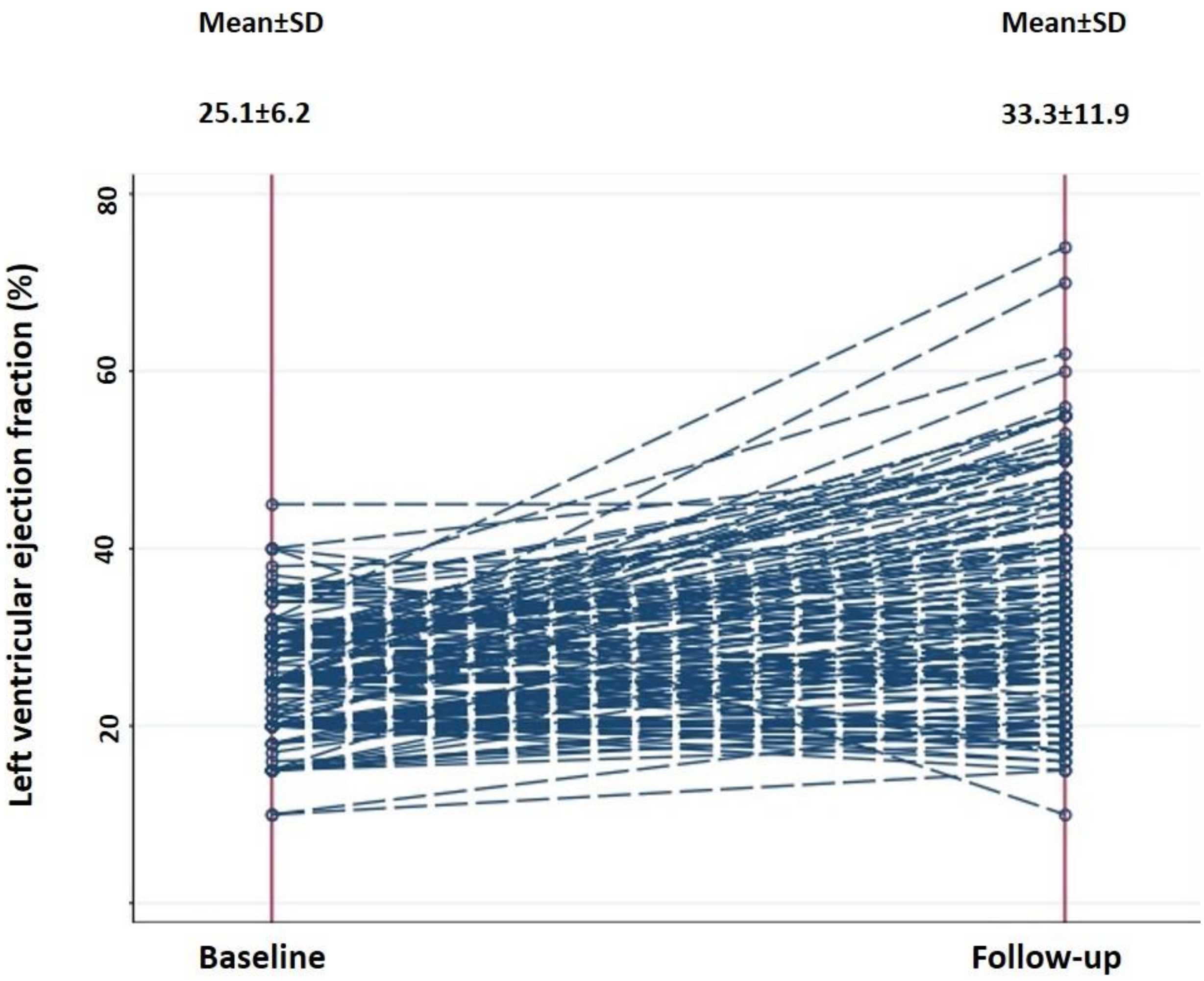

Supplement: Supplementary file 1 [file Datasheet1.pdf]
